# Sodium Thiosulfate Improves Hypertension in Rats with Adenine-Induced Chronic Kidney Disease

**DOI:** 10.3390/antiox11010147

**Published:** 2022-01-11

**Authors:** Chien-Ning Hsu, Chih-Yao Hou, Guo-Ping Chang-Chien, Sufan Lin, Hung-Wei Yang, You-Lin Tain

**Affiliations:** 1 Department of Pharmacy, Kaohsiung Chang Gung Memorial Hospital, Kaohsiung 833, Taiwan; cnhsu@cgmh.org.tw; 2 School of Pharmacy, Kaohsiung Medical University, Kaohsiung 807, Taiwan; 3 Department of Seafood Science, National Kaohsiung University of Science and Technology, Kaohsiung 811, Taiwan; chihyaohou@webmail.nkmu.edu.tw; 4 Center for Environmental Toxin and Emerging-Contaminant Research, Cheng Shiu University, Kaohsiung 833, Taiwan; guoping@csu.edu.tw (G.-P.C.-C.); linsufan2003@csu.edu.tw (S.L.); 5 Super Micro Mass Research and Technology Center, Cheng Shiu University, Kaohsiung 833, Taiwan; 6 Institute of Medical Science and Technology, National Sun Yat-sen University, Kaohsiung 804, Taiwan; 7 Department of Pediatrics, Kaohsiung Chang Gung Memorial Hospital and Chang Gung University College of Medicine, Kaohsiung 833, Taiwan; 8 Institute for Translational Research in Biomedicine, Kaohsiung Chang Gung Memorial Hospital and Chang Gung University College of Medicine, Kaohsiung 833, Taiwan

**Keywords:** symmetric dimethylarginine, chronic kidney disease, hydrogen sulfide, hypertension, nitric oxide, renin–angiotensin system, sodium thiosulfate

## Abstract

Hypertension is highly prevalent in chronic kidney disease (CKD). Hydrogen sulfide (H_2_S) is an endogenously produced gasotransmitter with vasodilator properties. We, hence, investigated whether oral administration of sodium thiosulfate (STS), a clinically applicable H_2_S-based therapy, can exert a protective effect against hypertension in an adenine-induced CKD rat model. Eight-week-old male Sprague–Dawley rats were fed with 0.5% adenine chow for 3 weeks to induce CKD. After 1 week, the rats were divided into two groups: one without and one with STS (2 g/kg body weight/day) in drinking water for 2 weeks. Treatment with STS lowered systolic and diastolic blood pressure by 7 and 9 mm Hg, respectively. Renal H_2_S-generating enzyme expression was inhibited by CKD, while STS therapy increased plasma levels of H_2_S and thiosulfate. Additionally, restoration of nitric oxide bioavailability and rebalance of the renin–angiotensin system may contribute to the protective effects of STS. Our data suggest that the oral administration of STS improves hypertension in an adenine-induced CKD model, which brings us closer to the clinical translation of H_2_S-targeting therapy in CKD-induced hypertension.

## 1. Introduction

Hydrogen sulfide (H_2_S) is a gasotransmitter, which plays an important role in several biological functions, including the renal regulation of blood pressure (BP) [1,2]. Hypertension and chronic kidney disease (CKD) both are common diseases all over the world [3], being closely connected to each other. Emerging evidence suggests that a reduction in H_2_S synthesis may be a key mechanism underlying hypertension and kidney disease [1,2,4]. In the kidneys, a mismatch of vasodilators, such as H_2_S and nitric oxide (NO), and vasoconstrictors, such as angiotensin II (Ang II), in favor of the latter, may ultimately lead to hypertension [5,6]. Conversely, H_2_S-based modalities have been developed for therapeutic protection against hypertension and kidney disease [6,7].

Enzymatic production of H_2_S from the substrate L-Cysteine occurs through three enzymes: cystathionine-lyase (CSE), cystathionine-synthase (CBS), and 3-mercaptopyruvate sulfurtransferase (3MST) [1]. In an alternative pathway, H_2_S is produced by cysteine amino transferase (CAT) and D-amino acid oxidase (DAO). Our prior work showed that dietary supplementation with L-Cysteine (the precursor of H_2_S) protects spontaneously hypertensive rats (SHRs) against high-salt-induced hypertension and kidney damage via the reduction of oxidative stress, mediation of the H_2_S-generating pathway, and restoration of the renin–angiotensin system (RAS) [8].

In addition, H_2_S can be non-enzymatically produced from organic thiol. Thiosulfate, an intermediate in oxidative H_2_S metabolism, can alternatively be reduced and regenerate H_2_S [9]. Although thiosulfate has shown therapeutic potential in hypertensive renal disease in a model of NO deficiency [10], limited information is available about whether thiosulfate therapy can protect against hypertension in CKD.

The adenine diet model results in progressive kidney injury and several complications, truly mimicking the pathophysiology of human CKD [11]. Our prior work illustrated that rats receiving chow supplemented with 0.5% adenine for 3 weeks develop CKD, characterized as renal dysfunction, glomerular and tubulointerstitial damage, hypertension, and increased uremic toxin levels [12]. The objective of this study reported here was to examine whether sodium thiosulfate (STS) treatment can protect against CKD-induced hypertension and elucidate the underlying mechanism using an adenine-induced CKD model.

## 2. Materials and Methods

### 2.1. Animal Model and Care

Male Sprague–Dawley (SD) rats (*n* = 32) were obtained from BioLASCO Taiwan Co., Ltd. (Taipei, Taiwan). On arrival, the rats were maintained in our animal facility awarded full accreditation from AAALAC International. The procedures used in this study were according to the rules of the Care and Use of Laboratory Animals of the National Institutes of Health and the IACUC of Chang Gung Memorial Hospital (Permit # 2020073101).

Afterward, each rat was randomly assigned to one of four treatments (*n* = 8 per group): normal diet (ND), diet supplemented with 0.5% adenine from 8 weeks of age for 3 weeks (CKD), normal diet with STS (2 g/kg body weight/day) in drinking water from 9 weeks of age for 2 weeks (ND + STS), and diet supplemented with 0.5% adenine from weeks 8 to 11 and STS from weeks 9 to 11 (CKD + STS). The doses of adenine and STS used here are based on previous studies conducted on rats [10,11]. We used the CODA rat tail-cuff system (Kent Scientific Corporation, Torrington, CT, USA) to measure BP [12]. All rats were acclimated to restraint and tail-cuff inflation for 1 week before the initiation of the experiment to ensure reproducibility and accuracy. All rats were sacrificed at 11 weeks of age. Blood samples were collected in tubes containing heparin. The kidneys were immediately snap-frozen and saved at −80 °C until analysis.

### 2.2. Analysis of Plasma Hydrogen Sulfide and Thiosulfate Levels

According to our validated protocol [13], concentrations of H_2_S and thiosulfate in the plasma were determined by an Agilent Technologies 1290 high-performance liquid chromatography (HPLC) system connected to an Agilent 6470 Triple Quadrupole LC/Mass Spectrometry (MS) (Agilent Technologies, Wilmington, DE, USA). We added internal standard phenyl 4-hydroxybenzoate (PHB) to the extraction solvent. We detected thiosulfate derivative pentafluorobenzyl (PFB)-S_2_O_3_H and H_2_S derivative sulfide dibimane (SDB). Selected reaction monitoring mode was used to detect target compounds with a targeted *m/z* 212.99→93, *m/z* 415→223, and *m/z* 292.99→81 for PHB, SDB, and PFB-S_2_O_3_H, respectively.

### 2.3. Quantitative Real-Time Polymerase Chain Reaction (qPCR)

Rat kidney cortex tissue was homogenized in lysis buffer, and total RNA was extracted using the TRIZOL method (Invitrogen, Carlsbad, CA, USA), as described earlier [13]. Two-step quantitative real-time PCR was conducted in duplicate using the QuantiTect SYBR Green PCR Kit (Qiagen, Valencia, CA, USA) on an iCycler iQ Real-Time PCR Detection System (Bio-Rad, Hercules, CA, USA). A total of four genes involved in H_2_S production were determined: *Cbs* (encoding for protein CBS), *Cth* (CSE), *Mpst* (3MST), and *Dao* (DAO). We also determined the renal expression of the genes belonging to the RAS, including *Ren* (renin), *Ace* (angiotensin converting enzyme-1), *Agtr1a* (angiotensin II type 1 receptor), *Ace2* (angiotensin converting enzyme-2), *Agtr2* (angiotensin II type 2 receptor), and *Mas1* (angiotensin (1–7) receptor MAS). *Rn18s* was used as the internal control as it was expressed at a constant level across the sample set. The comparative threshold cycle (Ct) method (2^−ΔΔCt^), represented as x-fold expression, was used to compare the mRNA levels. Table 1 provides the PCR primer sequences.

### 2.4. Western Blot

As previously described [8], kidney cortex samples were subjected to Western blotting with antibody incubation. For each sample, equal quantities of total protein (200 μg) were loaded and electrophoresed through a 10% polyacrylamide gel. Separated proteins were transferred into a nitrocellulose membrane (GE Healthcare Bio-Sciences Corp., Piscataway, NJ, USA). We used Ponceau S staining (PonS) to correct for variations in the total protein loading in the Western blot. After transfer, the nitrocellulose membranes were rinsed briefly in distilled water and incubated in 15 mL of a Ponceau S solution (0.2% (*w*/*v*) in 1% (*v*/*v*) acetic acid; Sigma-Aldrich, St. Louis, MO, USA) for 10 min on the rocker, followed by a brief rinse in distilled water so that the lanes and bands were clearly visible. The membranes were then scanned and the TIFF file saved for later quantification. After that, the membranes were incubated in distilled water for two washes of 5 min each and we proceeded with the blocking. The membranes were blocked in phosphate-buffered saline-Tween (PBS-T) containing 5% nonfat milk. For the detection of H_2_S-generating enzymes, the membranes were incubated with the following antibodies: a rabbit monoclonal anti-rat 3MST antibody (1:500, overnight incubation; Novus Biologicals, Littleton, CO, USA), a mouse monoclonal anti-rat CBS antibody (1:1000, overnight incubation; Abnova Corporation, Taipei, Taiwan), or a rabbit polyclonal anti-rat CSE antibody (1:1000, overnight incubation; Proteintech Group, Inc. Chicago, IL, USA). The bands were detected using an enhanced chemiluminescence reagent (PerkinElmer, Waltham, MA, USA). The abundance of protein expression was quantified by densitometry (Quantity One Analysis software, Bio-Rad) and calculated as the integrated optical density (IOD) in relation to the background value. The relative protein abundance was expressed as IOD/PonS to correct the variations in total protein loading.

### 2.5. Determination of Nitric Oxide Parameters

We used the HP Agilent 1100 HPLC System (Agilent Technologies Inc., Santa Clara, CA, USA) with fluorescence detection of O-phthalaldehyde/3-mercaptopropionic acid (OPA/3-MPA) derivatives to measure NO-related parameters in the plasma. These parameters include L-Arginine, L-Citrulline (the precursor of L-Arginine), and NO synthase inhibitor asymmetric and symmetric dimethylarginine (ADMA and SDMA). The standards contained 1–100 mM L-Citrulline, 1–100 mM L-Arginine, 0.5–5 mM ADMA, and 0.5–5 mM SDMA. L-Arginine was divided by ADMA plasma levels for calculating the L-Arginine-to-ADMA ratio, a determinant of NO bioavailability [14].

### 2.6. Statistical Analysis

All data are presented as the mean ± the standard error of the mean. Statistical analyses were performed using one-way ANOVA or two-way ANOVA where appropriate. Tukey’s post hoc test was applied where multiple comparisons were made. *p* < 0.05 was considered significant. Statistical analyses were performed using SPSS software (SPSS Inc., Chicago, IL, USA).

## 3. Results

### 3.1. Body Weight and Blood Pressure of Male Offspring

The body weight (BW) of the CKD and CKD + STS groups was lower, while the kidney weight (KW) and the KW-to-BW ratio were higher compared to those of the control (Table 2). Baseline BP values were not significantly different among groups. At 11 weeks of age, the CKD group showed increases in systolic and diastolic BP and the mean arterial pressure was significantly attenuated in the CKD + STS group. Taken together, these findings indicate that CKD-induced hypertension is improved by STS treatment.

### 3.2. H_2_S Pathway

**Figure 1:**
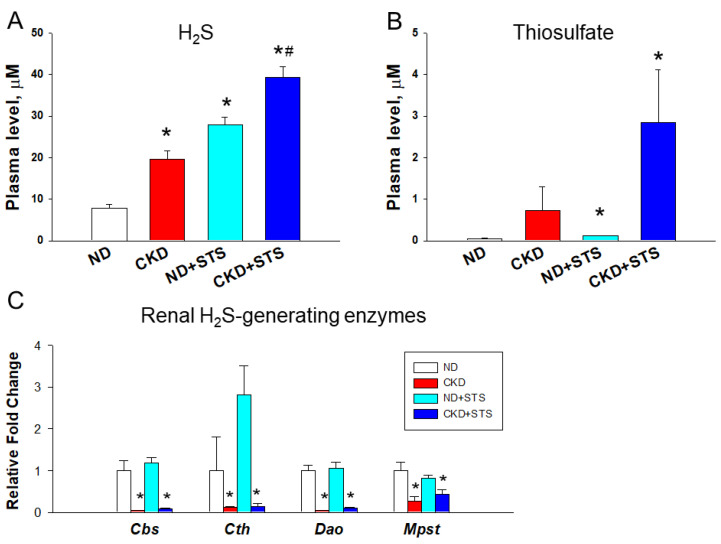
Effects of sodium thiosulfate (STS) on the (**A**) plasma H_2_S level, (**B**) plasma thiosulfate level, and (**C**) renal mRNA expression of H_2_S-generating enzymes. All the results represent the mean ± the standard errors of eight animals in each group. Data are analyzed by one-way ANOVA followed by Tukey’s post hoc test. * *p* < 0.05 versus control g; * *p* < 0.05 vs. ND; # *p* < 0.05 CKD.

Results for the H_2_S pathway are shown in Figure 1, which include plasma H_2_S and thiosulfate levels and the renal mRNA expression of H_2_S-generating enzymes. Compared to ND, the plasma H_2_S level was higher in the other three groups (Figure 1A). The STS treatment showed a further increased H_2_S level in the CKD + STS group compared to the CKD group. Similarly, an increased plasma thiosulfate level was observed in the ND + STS and CKD + STS groups compared to the control (Figure 1B). Renal transcript abundance of H_2_S-generating enzymes *Cbs, Cth, Dao*, and *Mpst* is compared in Figure 1C. Compared to ND, four H_2_S-generating enzymes showed similar downregulation patterns in the CKD and CKD + STS groups.

**Figure 2:**
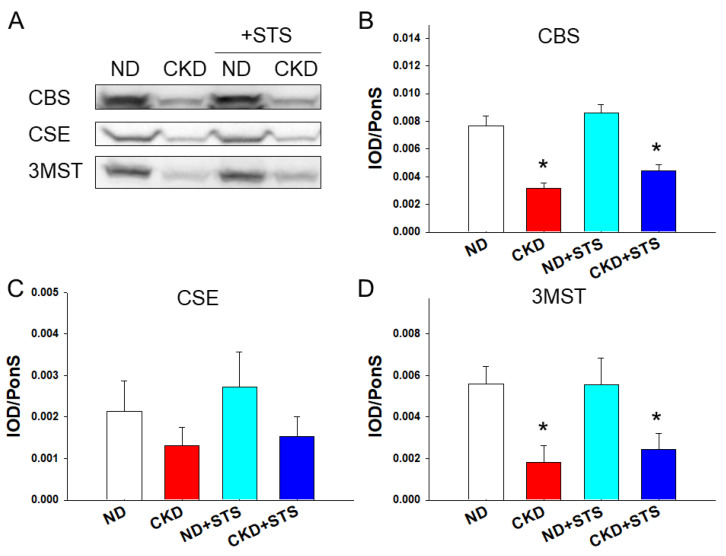
Effects of sodium thiosulfate (STS) on H_2_S-generating enzymes in the kidneys. (**A**) Representative Western blots show cystathionine β-synthase (CBS, ~61 kDa), cystathionine γ-lyase (CSE, ~45 kDa), and 3-mercaptopyruvate sulfurtransferase (3MST, ~52 kDa) bands. The relative abundance of renal cortical (**B**) CBS, (**C**) CSE, and (**D**) 3MST was quantified. All the results represent the mean ± the standard errors of eight animals in each group. Data are analyzed by one-way ANOVA followed by Tukey’s post hoc test. * *p* < 0.05 vs. ND.

Renal protein abundance of H_2_S-generating enzymes is compared in Figure 2. CKD significantly reduced renal CBS and 3MST protein levels in the CKD and CKD + STS groups (Figure 2B,D), while STS treatment had a negligible effect on their levels. Although CKD showed a similar tendency to the renal CSE protein abundance, it did not reach the significance (Figure 2C). Collectively, these findings indicate that STS treatment increases plasma H_2_S and thiosulfate levels without affecting renal H_2_S-generating enzyme expression.

### 3.3. NO Pathway

Plasma NO parameters are compared in Table 3. Compared to ND, plasma L-Citrulline and L-Arginine levels were lower in the other three groups. The CKD group had higher plasma ADMA levels, but thiosulfate reduced this effect. The STS treatment reduced SDMA in the ND + STS group. In addition, CKD only reduced the L-Arginine-to-ADMA ratio in rats with CKD. These results therefore indicate that in rats with CKD, the NO pathway is impaired, characterized as increased ADMA but decreased L-Arginine and decreased L-Arginine-to-ADMA ratio. In contrast, decreased NO bioavailability was improved by STS therapy.

### 3.4. Renin-Angiotensin System

**Figure 3:**
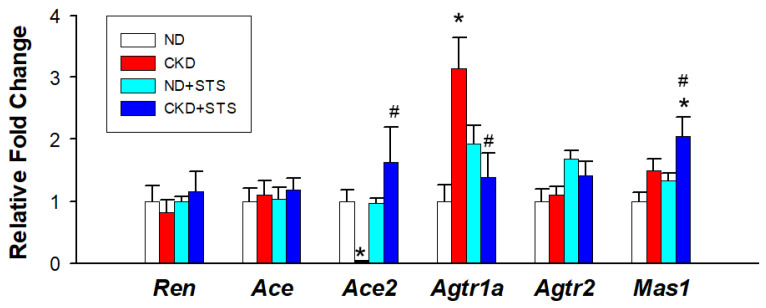
Effects of sodium thiosulfate (STS) on the renin–angiotensin system. All the results represent the mean ± the standard errors of eight animals in each group. Data are analyzed by one-way ANOVA followed by Tukey’s post hoc test. * *p* < 0.05 vs. ND; # *p* < 0.05 vs. CKD.

We further evaluated the RAS genes by qPCR (Figure 3). CKD reduced the renal mRNA expression of *Ace2*, which was restored by STS treatment. The STS treatment significantly reduced CKD-induced increases of renal *Agtr1a* expression. Additionally, *Mas1* expression was greater in the CKD + STS group compared with that in the CN and CKD groups.

## 4. Discussion

This work casts new light on the interaction between H_2_S signaling, the NO pathway, and the RAS in the kidney by which STS therapy prevents hypertension induced by CKD. The major contributions of our study are summarized as follows: (1) STS treatment attenuated the elevation of BP in rats with adenine-induced CKD, (2) CKD caused a reduction in renal H_2_S-generating enzyme expression, while STS treatment had a negligible effect on their levels, (3) STS therapy increased plasma levels of H_2_S and thiosulfate, (4) the beneficial effect of STS was associated with the reduction of ADMA and the restoration of L-Arginine-to-ADMA ratio in the plasma, and (5) STS treatment enhanced RAS gene *Ace2* and *Mas1* but decreased *Agtr1a*.

The findings of this research are in line with prior studies indicating that decreased renal H_2_S-generating enzyme protein levels and/or activity is involved in the pathogenesis of hypertension and CKD [15,16]. Although H_2_S exhibits a wide range of biological functions [1,2,17], limited data are available regarding the effects of STS on CKD-induced hypertension. According to our data, treatment with STS increased plasma levels of H_2_S and thiosulfate while it had little effect on renal H_2_S-generating enzymes.

The reduction in BP observed in this study is consistent with previous findings displaying the vasorelaxant properties of H_2_S [7,8,13]. H_2_S can be endogenously produced or exogenously delivered. We have previously shown that the administration of precursors of H_2_S, such as L-Cysteine or N-Acetylcysteine, could enhance endogenous H_2_S production and provide protection against hypertension [8,18]. Considering H_2_S-generating enzymes are inhibited by CKD in our model, this might not be an appropriate approach for CKD-induced hypertension. H_2_S donors have been evaluated as having the therapeutic potential of exogenous H_2_S [19]. However, exogenous H_2_S donors might be toxic whenever they cause a short-term increase in H_2_S to supraphysiological concentrations, which limit their clinical translation.

The current study provides another possibility in the use of sodium thiosulfate. As dynamic conversion exists between thiosulfate and H_2_S [9], thiosulfate can serve as a steady source of H_2_S in our body. Treatment with STS for 2 weeks in our study reduced SBP and DBP by 7 and 9 mmHg in rats with adenine-induced CKD, respectively. The BP lowering effect of STS in our work is similar to that previously reported in an NO deficiency model [10]. In the present study, the antihypertensive effects of STS were achieved in the face of increases in plasma thiosulfate and H_2_S. As STS has been used for the treatment of calciphylaxis and has a proven safety profile [20], our data have extended the possibility of using STS for CKD-induced hypertension for future clinical translation.

A key vasorelaxant action of H_2_S is to enhance NO signaling [1,17]. In our CKD model, the beneficial effect of STS is likely considering that it relies on the restoration of NO bioavailability. In the current study, the CKD-caused elevation of BP coincides with an increased ADMA level and a decreased ratio of L-Arginine to ADMA, a determinant of NO bioavailability. Conversely, these changes were restored by STS treatment. Accordingly, the protective effect of STS against hypertension is, at least in part, attributed to its ability to restore reduced NO bioavailability. However, the extent to which the organ-specific expression of NO synthase enzymes was affected by STS awaits further evaluation. Notably, previous studies have revealed cross-talk mechanisms between H_2_S, NO, and carbon monoxide (CO) against hypertension [4,6]. H_2_S can modify cysteine residues on key signaling molecules such as nuclear factor erythroid 2-related factor 2 (NRF2) and nuclear factor κB (NFκB), thereby promoting antioxidant and anti-inflammatory effects [6]. Activation of the NRF2-heme oxygenase-1 (HO-1)-CO signaling pathway can counterbalance oxidative stress [21]. NO can mediate NFκB to reduce inflammation [22]. Because of the cross-talk between the three gasotransmitters, there will be a growing need to better understand the possible contribution of NO and CO production to the observed beneficial effects of STS.

Another probable beneficial effect of STS is related to its ability to rebalance the RAS. Consistent with prior research indicating that the aberrant activation of the classic RAS is associated with CKD [23], our data demonstrate that CKD enhances renal AT1R expression. It is well known that the classical RAS can be activated by Ang II to trigger kidney damage and vasoconstriction via AT1R [24]. Reduction of AT1R expression by STS therapy may offset the detrimental effects of Ang II signaling in favor of vasodilatation. Conversely, STS treatment enhances ACE2 and MAS expression. Activation of the counter-regulatory ACE2–angiotensin (1–7)–MAS receptor pathway is able to induce vasodilatation, which may have a role in the beneficial effects of STS. Although H_2_S was reported to inhibit the activity of renin [25], we found that STS had little effect on its expression in this model. Collectively, these findings suggest that rebalance of the RAS might be involved in the protective effects of STS in our model.

There are still some limitations. One limitation of this study is that we did not analyze other organs that control BP. The protective effects of STS against hypertension may be attributed to other organ systems, such as the vasculature, the heart, or the brain. For future research, it would be interesting to conduct a thorough examination of aforementioned protective mechanisms of STS in an organ-specific manner. Second, we did not determine which cells in the kidney are involved in the expression of H_2_S-generating enzymes by immunohistochemistry. Another limitation is that we did not test other treatment doses or duration of STS. Whether STS treatment displays a dose- or time-dependent effect deserves further clarification. Although the use of STS has been approved for the treatment of calciphylaxis in patients with CKD [26], its safety and efficacy for hypertension await further evaluation prior to clinical translation. Lastly, we should notice that aforementioned mechanisms might not cover the whole picture of the protective benefits of STS treatment. Considering that gut microbiota are another source of H_2_S [17], additional studies are required to illuminate whether the gut microbiota are also involved in the protective role of STS against hypertension.

## 5. Conclusions

In conclusion, we demonstrated that the oral administration of STS improves hypertension in an adenine-induced CKD model, which brings us closer to clinical translation. Approaches targeting the H_2_S pathway might have therapeutic potential in CKD-induced hypertension.

## Figures and Tables

**Table 1:** PCR primer sequences.

Gene	Forward (5′–3′)	Reverse (5′–3′)
*Cbs*	atgctgcagaaaggcttcat	gtggaaaccagtcggtgtct
*Cth*	cgcacaaattgtccacaaac	gctctgtccttctcaggcac
*Mpst*	ggctcagtaaacatcccattc	tgtccttcacagggtcttcc
*Dao*	ccctttctggaaaagcacag	ctcctctcaccacctcttcg
*Ren*	aacattaccagggcaactttcact	acccccttcatggtgatctg
*Ace*	caccggcaaggtctgctt	cttggcatagtttcgtgaggaa
*Ace2*	acccttcttacatcagccctactg	tgtccaaaacctaccccacatat
*Agtr1a*	gctgggcaacgagtttgtct	cagtccttcagctggatcttca
*Agtr1b*	caatctggctgtggctgactt	tgcacatcacaggtccaaaga
*Mas*	catctctcctctcggctttgtg	cctcatccggaagcaaagg
*Rn18s*	gccgcggtaattccagctcca	cccgcccgctcccaagatc

**Table 2:** Weight and blood pressure.

Groups	ND	CKD	ND + STS	CKD + STS
Body weight (BW) (g)	374 ± 6	292 ± 7 *	377 ± 7	270 ± 12 *
Left kidney weight (g)	1.89 ± 0.09	3.54 ± 0.23 *	1.8 ± 0.07	2.94 ± 0.17 *
Left kidney weight/100 g BW	0.5 ± 0.006	1.21 ± 0.060 *	0.48 ± 0.019	1.09 ± 0.037 *
Systolic BP (mmHg)	129 ± 1	145 ± 2 *	132 ± 1	138 ± 1 #
Diastolic BP (mmHg)	86 ± 2	101 ± 3 *	90 ± 3	92 ± 2 #
Mean arterial pressure (mmHg)	100 ± 1	115 ± 2 *	104 ± 2	107 ± 1 #

*n* = 8/group; * *p* < 0.05 vs. ND; # *p* < 0.05 CKD + STS vs. CKD; BP = blood pressure.

**Table 3:** Plasma NO parameters.

Groups	ND	CKD	ND + STS	CKD + STS
l-Citrulline (μM)	117.3 ± 7	101.3 ± 5.3 *	83.8 ± 6.8 *	99.1 ± 3.9 *
l-Arginine (μM)	333.2 ± 15.5	236.8 ± 11.2 *	231.7 ± 28.4 *	190.1 ± 17.8 *#
Asymmetric dimethylarginine (μM)	1.51 ± 0.17	1.96 ± 0.12 *	1.01 ± 0.18	1.21 ± 0.09 #
Symmetric dimethylarginine (μM)	1.18 ± 0.07	1.21 ± 0.13	0.81 ± 0.09 *	1.07 ± 0.18
l-Arginine-to-ADMA ratio (μM/μM)	242 ± 30	125 ± 11 *	254 ± 29	167 ± 23

All the results represent the mean ± the standard errors of eight animals in each group. Data are analyzed by one-way ANOVA followed by Tukey’s post hoc test. * *p* < 0.05 vs. ND; # *p* < 0.05 vs. CKD.

## Data Availability

Data are contained within the article.
